# Multimodality imaging evaluation of nasal sinus alveolar rhabdomyosarcoma: Two case reports

**DOI:** 10.3389/fmed.2022.1047464

**Published:** 2022-11-10

**Authors:** Wenpeng Huang, Yongbai Zhang, Ge Gao, Liming Li, Qi Yang, Yongkang Qiu, Lei Kang

**Affiliations:** ^1^Department of Nuclear Medicine, Peking University First Hospital, Beijing, China; ^2^Department of Medical Imaging, Peking University First Hospital, Beijing, China; ^3^Department of Radiology, The First Affiliated Hospital of Zhengzhou University, Zhengzhou, China

**Keywords:** alveolar rhabdomyosarcoma, 18F-FDG, PET/CT, X-ray computed tomography, magnetic resonance imaging, case report

## Abstract

**Background:**

Rhabdomyosarcoma (RMS) is a common pleomorphic malignant soft tissue sarcoma in children and adolescents that originates from rhabdomyoblasts or mesenchymal precursor cells. Alveolar rhabdomyosarcoma (ARMS) mostly occurs in adolescents aged 10–15 years and is characterized by more aggressive behaviors and worse prognosis than other sarcomas, prone to lymphatic and hematogenous metastasis in the early stage as well as metastasizing to breast, testis, pancreas, and other parts. ARMS often occurs in the limbs and genitourinary system, however, head and neck ARMS are relatively rare when involving the nasal cavity or sinuses. The role of MRI and ^18^F-FDG positron emission tomography combined with computed tomography (PET/CT) remains to be established in ARMS.

**Case report:**

Case 1: An 18-year-old male was found with a left submandibular mass of approximately 1 cm in diameter 2 months ago, which gradually increased in size. CT showed multiple soft tissue masses in maxillofacial and neck regions and the lesions invaded the frontal lobe and the inner wall of the left orbital lobe. MRI showed the masses with hypointensity on T1WI, hyperintensity on T2WI, and diffusion-weighted imaging (DWI) with significant enhancement. ^18^F-FDG PET/CT showed multiple hypermetabolic lesions located in the maxillofacial, neck region, 3rd lumbar vertebra, and the right sacrum. A nasal endoscopic tumor biopsy and molecular testing finally helped to diagnose the ARMS. Case 2: A 14-year-old male presented with left maxillary pain with nasal congestion and left ocular swelling 15 days ago. CT demonstrated a soft tissue mass in the nasal cavity and sinuses with local protrusion into the left orbit. MRI showed the masses with a slightly low signal on T1WI, a high signal on T2WI, and DWI with significant heterogenous enhancement. ^18^F-FDG PET/CT showed hypermetabolic lesions in the left maxillofacial and neck regions. ARMS was finally diagnosed by a nasal endoscopic tumor biopsy and molecular testing. The patient had a recurrence of the lesion after chemotherapy and surgical resection and is currently undergoing radiation therapy.

**Conclusion:**

Nasal sinus ARMS is highly malignant with a poor prognosis. Accurate diagnosis relies not only on histopathology and immunohistochemistry examination but also on genetic detection of characteristic chromosomal translocations and fusion genes. Imaging methods, such as MRI and PET/CT can accurately assess the extent of the lesions and metastases, assist in the diagnosis of the disease and the selection of treatment regimens, provide precise localization for surgery, and help with treatment monitoring and follow-up.

## Introduction

Rhabdomyosarcoma (RMS) is a common pleomorphic malignant soft tissue sarcoma in children and adolescents that originates from rhabdomyoblasts or mesenchymal precursor cells (1). Alveolar rhabdomyosarcoma (ARMS) was first reported by Enziger and Shiraki in 1969 (2). It mostly occurs in adolescents aged 10–15 years and is characterized by more aggressive behaviors and a worse prognosis than other sarcomas, prone to lymphatic and hematogenous metastasis in the early stage as well as metastasizing to breast, testis, pancreas, and other parts (3, 4). ARMS often occurs in the limbs and genitourinary system, however, head and neck ARMS are relatively rare when involving the nasal cavity or sinuses (5, 6).

Nasal endoscopy, imaging examination, and biopsy pathology are the main diagnostic methods for nasal sinus tumors. The structure of the nasal cavity and sinuses is special and includes mainly bony structures, covered mucosa, and air in the cavity. Computed tomography (CT) and magnetic resonance imaging (MRI) are often used in combination to observe the characteristics, blood supply, and extent of lesions, especially for lesions located in deeper locations, and the combination of nasal endoscopy and imaging can improve the diagnostic accuracy of biopsy pathology. It is an imaging technology that reveals detailed metabolic and functional molecular information as well as the precise site of the lesion using 2-Deoxy-2-[fluorine-18]-fluoro-D-glucose (^18^F-FDG) positron emission tomography combined with computed tomography (PET/CT). It has been used to evaluate a variety of tumors and represents an extremely promising investigation method for RMS diagnosis, staging, and prognosis. Here, we report multiple imaging characteristics of two cases with nasal sinus ARMS and discuss the clinical and imaging features to improve the understanding of this type of disease.

## Case presentation

### Case 1

**FIGURE 1:**
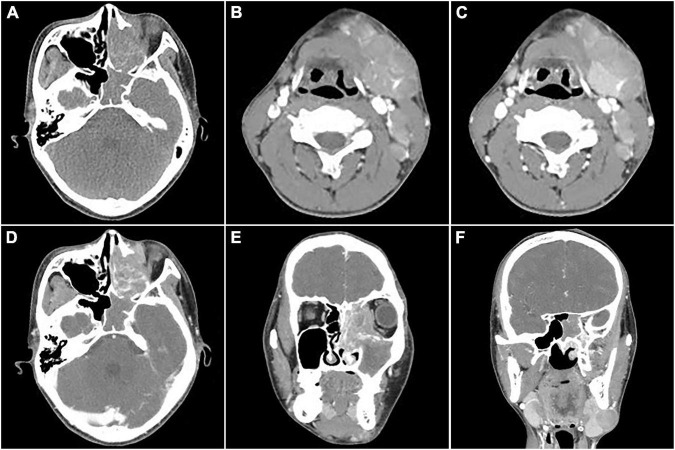
Computed tomography (CT) images of nasal sinus alveolar rhabdomyosarcoma (ARMS) in case 1. **(A)** Plain image showed the lesion at the left nasal cavity and sphenoidal sinus with homogenous density and osteolytic destruction inside. **(B)** The arterial phase contrast image showed circular enhancement in the left mandibular and cervical masses. (**C–F**; **C,D**, transverse; **E,F**, coronal) Venous phase images showed bilateral lesions in the neck and parapharyngeal regions with heterogenous enhancement, which invaded the inner wall of the left orbit, the ethmoid inward, and the frontal lobe upward.

**FIGURE 2:**
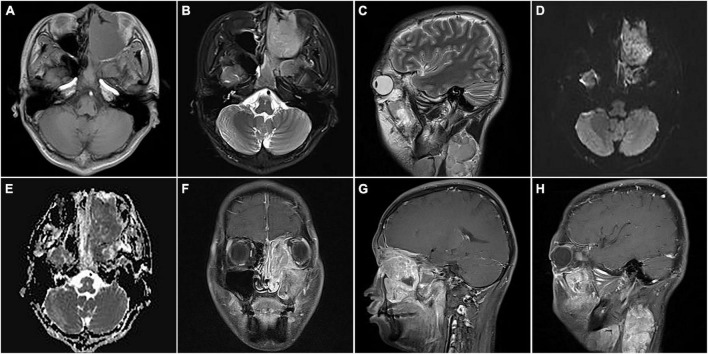
Magnetic resonance images of nasal sinus ARMS in case 1. **(A)** Transverse T1-weighted image (T1WI) showed the lesions at the left nasal cavity and nasal sinus with hypointensity. **(B)** Transverse T2-weighted image (T2WI) showed the lesions at the left nasal cavity and nasal sinus with homogenous hyperintensity. **(C)** Sagittal T2WI showed a cervical lesion with heterogenous hyperintensity. The lesions showed hyperintensity on diffusion-weighted imaging (DWI) **(D)** and hypointensity apparent diffusion coefficient (ADC) images **(E)**. Coronal **(F)** and sagittal **(G)** enhanced images showed lesions in the nasal sinus with distinct enhancement. **(H)** Sagittal-enhanced images showed cervical lesions with distinct enhancement and clear boundaries.

**FIGURE 3:**
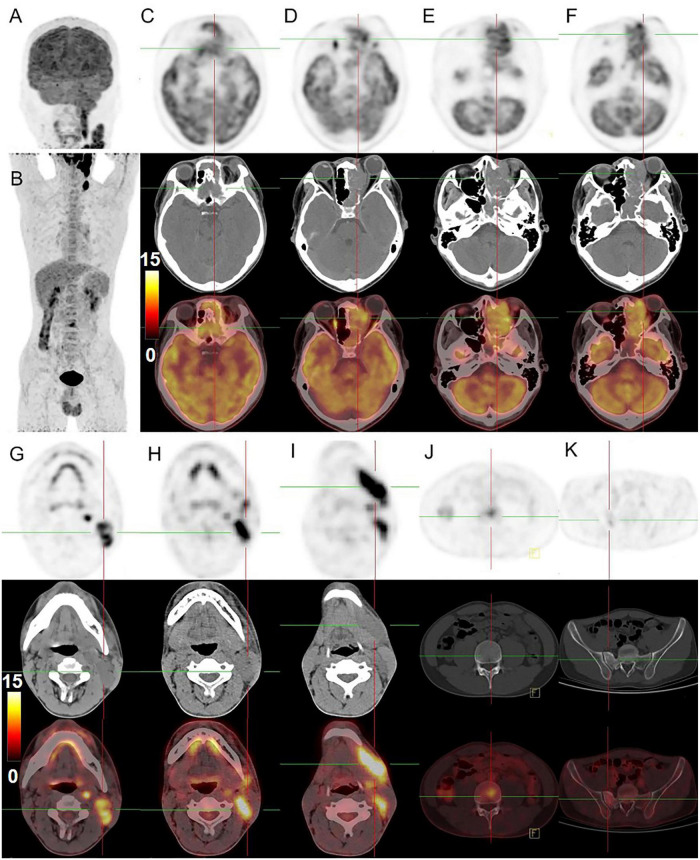
^18^F-FDG PET/CT images of nasal sinus ARMS in case 1. **(A,B)** The anteroposterior 3-dimensional maximum intensity projection image (MIP) demonstrated hypermetabolic lesions in the maxillofacial, neck region, 3rd lumbar vertebra, and the right sacrum. **(C–F)** The transverse images showed bilateral frontal sinus and left ethmoidal sinus, sphenoidal sinus, maxillary sinus, and nasal cavity filled with soft tissue shadows, with increased FDG uptake at a SUVmax of 12.6 and maximum section of about 4.1 cm × 4.6 cm. The lesion was shown with unclear boundaries from the adjacent structures. **(G–I)** The transverse images showed multiple enlarged lymph nodes in the left parapharyngeal regions and left cervical regions I-IV with significantly higher uptake of SUVmax about 18.6, a larger one was measured at 1.8 cm × 1.6 cm. **(J,K)** The transverse images showed the 3rd lumbar vertebra and the right sacrum with dense foci of radioactive distribution, SUVmax of about 5.2.

**FIGURE 4:**
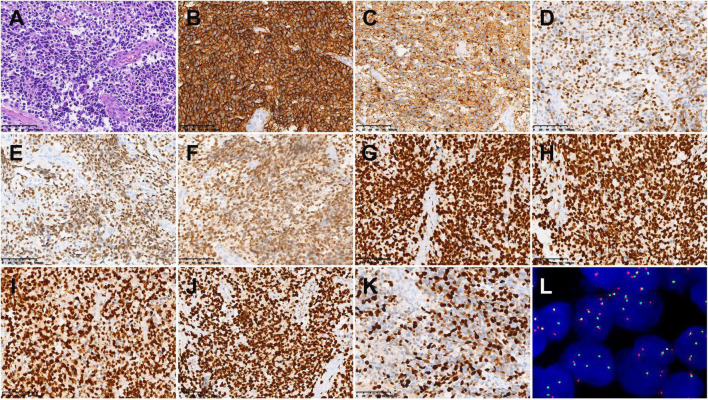
Histopathological results in case 1. **(A)** Hematoxylin-eosin (HE) staining (×200). Immunohistochemistry showed that the tumor cells were positive for CD56 **(B)**, Syn **(C)**, SOX-10 **(D)**, MyoD1 **(E)**, Myogenin **(F)**, MSH2 **(G)**, MSH6 **(H)**, MLH1 **(I)**, PMS2 **(J)**, and MSH6 **(K)** [Envision **(B–K)** ×200]. **(L)** Fluorescence *in situ* hybridization revealed *FKHR* gene rupture (×1,000).

An 18-year-old male was found with a left submandibular mass of approximately 1 cm in diameter 2 months ago, which gradually increased in size and volume. The patient presented with lacrimation in the left eye 1 month ago, an occipital headache 20 days ago, and swelling and protrusion of the left eye 1 week ago. Physical examination revealed multiple enlarged lymph nodes in regions I and II of the left side of the neck which were fused with poor range of motion, while the largest was measured at approximately 3.0 cm × 2.0 cm. Laboratory tests showed no abnormalities in tumor markers. CT showed multiple soft tissue masses in the left nasal cavity, sphenoidal sinus, frontal sinus, maxillary sinus, ethmoidal sinus, and bilateral mandible, neck, and parapharyngeal regions, with a maximum cross-section of about 5.9 cm × 2.5 cm. Contrast-enhanced CT revealed the masses with circular enhancement and the lesions invaded the frontal lobe (Figure 1). MRI showed multiple masses of abnormal signals in the above areas, with hypointensity on T1WI, hyperintensity on T2WI and diffusion-weighted imaging (DWI), and hypointensity on apparent diffusion coefficient (ADC) images, while the lesions showed significant enhancement on enhanced images (Figure 2). Then, the patient was injected with 0.1 mCi/kg of ^18^F-FDG after 6 h of fasting and PET/CT images were acquired 60 min later for further whole-body evaluation. Bilateral frontal sinus and left ethmoidal sinus, sphenoidal sinus, maxillary sinus, and nasal cavity were filled with soft tissue, with increased FDG uptake at a maximum standardized uptake value (SUVmax) of 12.6 at about 4.1 cm × 4.6 cm. The lesion showed an unclear boundary from the frontal lobe, and protrusion into the left orbital lobe whereas the left abducens oculi and deprimens oculi were displaced by pressure with the eyeball protruding anteriorly. The left maxillary sinus wall, turbinate, nasal septum, ethmoidal-lamina papyracea, sphenoidal sinus wall, frontal sinus wall, and clivus were invaded. Multiple enlarged lymph nodes were revealed in left parapharyngeal regions and left cervical regions I-IV with significantly high uptake of SUVmax at 18.6 and the largest one measuring 1.8 cm × 1.6 cm. In the right cervical regions II and IV, multiple slight-enlarged lymph nodes were displayed with SUVmax at about 3.2 and the largest one measuring 0.7 cm × 1.1 cm. The 3rd lumbar vertebra and the right sacrum were found with a local FDG-avid lesion with SUVmax at about 5.2 (Figure 3). A nasal endoscopic tumor biopsy was then performed to confirm the diagnosis. Pathological results showed that the tumor cells were round and oval with hyperchromatic nuclei and nuclear fission and formed a characteristic structure resembling pulmonary alveoli with fibrovascular intervals between alveoli. Immunohistochemistry showed CK (partial+), EMA (−), CD56 (+), Syn (+), TTF-1 (−), CD20 (−), CD3 (−), S-100 (partial+), SOX-10 (+), GFAP (−), P63 (−), P40 (−), NSE (−), Desmin (partial+), MyoD1 (+), Myogenin (+), CD30 (−), PD-1 (−), MSH2 (+), MSH6 (+), MLH1 (+), PD-L1 (−), PMS2 (+), and Ki-67 (70%+). Fluorescence *in situ* hybridization revealed *fork head* in RMS (FKHR) gene rupture. Based on the results of histological morphology, immunohistochemistry, and molecular testing, this case was finally diagnosed with ARMS (Figure 4). The patient refused radiation therapy and died 7 months after leaving the hospital.

### Case 2

**FIGURE 5:**
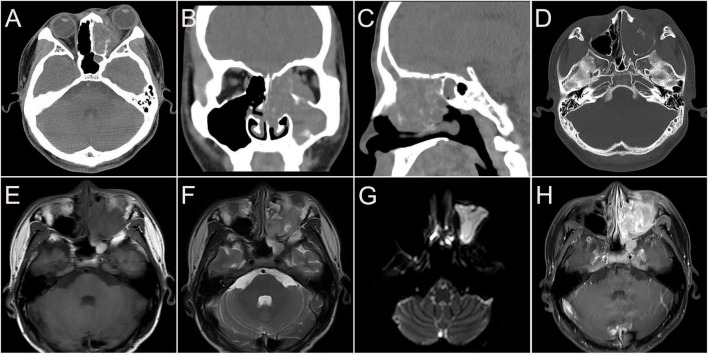
CT and MR images of nasal sinus ARMS in case 2. **(A–D)** CT images (**A**. transverse, **B**. coronal, **C**. sagittal, and **D**. bone window) showed soft tissue mass in the left nasal cavity, maxillary sinus, ethmoidal sinus, sphenoidal sinus, frontal sinus, and nasal cavity with local protrusion into the left orbit, interrupted bony continuity of the medial wall of the left maxillary sinus and lateral wall of the septal sinus. **(E)** The lesions showed slightly low signal T1WI. **(F)** High signal on T2WI. **(G)** High signal on DWI. **(H)** Significant heterogenous enhancement on enhanced images.

**FIGURE 6:**
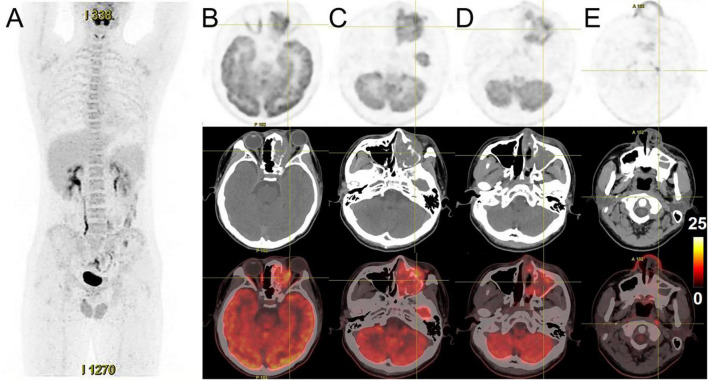
^18^F-FDG PET/CT images of nasal sinus ARMS in case 2. **(A)** The anteroposterior 3-dimensional MIP image demonstrated FDG-avid lesions located in the left maxillofacial region, without abnormal FDG metabolism. **(B–D)** The transverse images showed soft tissues existed in the left ethmoidal sinus, the left maxillary sinus, and the left nasal cavity, with high radioactivity distribution, SUVmax at about 19.1. The lesions invaded the left orbit without clear edges from the left abducens oculi, and bone destruction could be seen in the left ethmoidal-lamina papyracea, medial orbital wall, and maxillary sinus. **(E)** The transverse images showed a lymph node with elevated uptake of SUVmax at about 9.7 in the left parapharyngeal regions.

**FIGURE 7:**
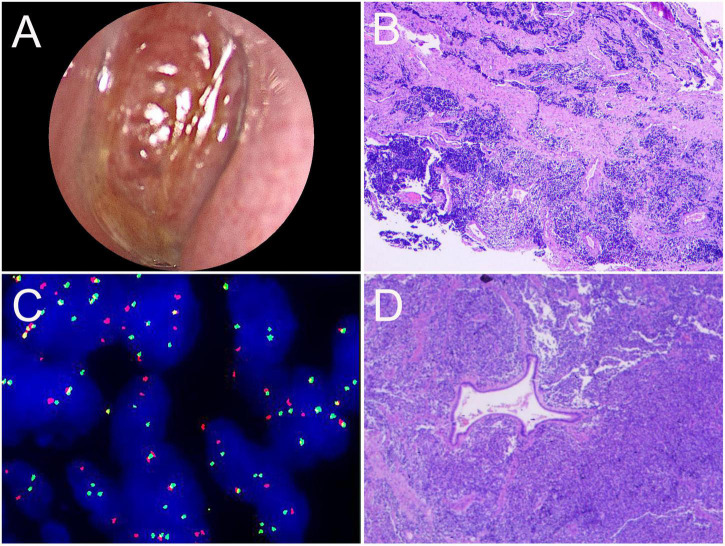
Nasal endoscopic images of the nasal sinuses and histopathological results of ARMS in case 2. **(A)** Nasal endoscopy of the patient revealed a reddish neoplasm in the left middle nasal tract. **(B)** HE staining of biopsy tissue (×40). **(C)** The gene test image showed positive *FKHR* gene rupture (×1,000). **(D)** Postoperative pathological HE staining (×100).

A 14-year-old male presented with left maxillary pain with nasal congestion and left ocular swelling 15 days ago. No obvious abnormality was observed in the laboratory examination. CT demonstrated a soft tissue mass in the left nasal cavity, maxillary sinus, ethmoidal sinus, sphenoidal sinus, frontal sinus, and nasal cavity with the local protrusion into the left orbit interrupted bony continuity of the medial wall of the left maxillary sinus and lateral wall of the septal sinus (Figures 5A–D). MRI showed multiple masses of abnormal heterogeneous signal in these areas, with slightly low signal on T1WI, high signal on T2WI and DWI, and significant heterogenous enhancement on enhanced images. The lesion could not be separated from the left abducens oculi and orbital wall, with compression of the left optic nerve and protrusion of the left eyeball (Figures 5E–H). ^18^F-FDG PET/CT demonstrated that soft tissues existed in the left ethmoidal sinus, the left maxillary sinus, and the left nasal cavity, with significant radioactive uptake of SUVmax at 19.1. The lesions invaded the left orbit without clear edges from the left abducens oculi, while bone destruction could be seen in the left ethmoidal-lamina papyracea, medial orbital wall, and maxillary sinus. Lymph nodes were revealed with elevated uptake of SUVmax at 9.7 in the left parapharyngeal regions, measuring about 0.5 cm × 0.7 cm (Figure 6). Nasal endoscopy of the patient revealed a neoplasm in the left middle nasal tract (Figure 7A). Biopsy of the lesion showed round and ovoid tumor cells with eosinophilic cytoplasm, heterogeneous deep-stained nuclei, and active nuclear division (Figure 7B). Immunohistochemistry showed CK (−), EMA (−), CD56 (+), CgA (−), SYN (+), S-100 (little+), CD99 (−), FLI-1 (partial+), Desmin (+), SMA (−), Myogenin (partial+), Myo D1 (+), ALK (+), and Ki-67 (80%+). Fluorescence *in situ* hybridization showed that FKHR gene rupture occurred in this case (Figure 7C). Therefore, an ARMS was finally diagnosed.

**FIGURE 8:**
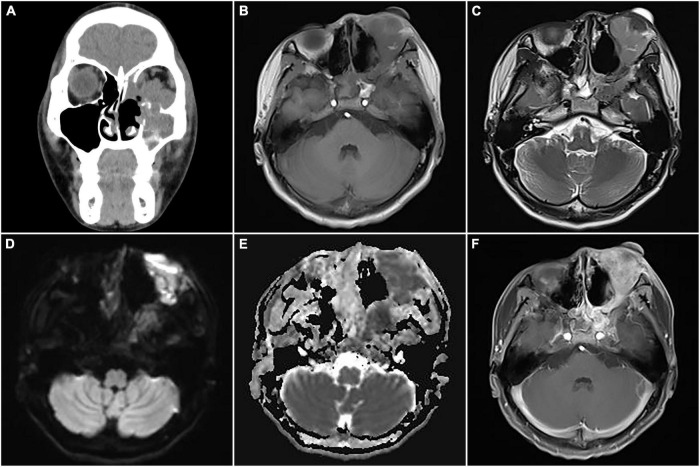
Computed tomography (CT) images and magnetic resonance imaging (MRI) of postoperative recurrence of alveolar rhabdomyosarcoma (ARMS) in case 2. **(A)** A coronal CT image showed soft tissue density masses in the sinuses, suggesting recurrence. **(B)** The lesions showed slight hypointensity on T1WI. **(C)** Slight hyperintensity on T2WI. **(D)** Hyperintensity on DWI. **(E)** Hypointensity on apparent diffusion coefficient (ADC) image. **(F)** Significant heterogeneous enhancement and compressed left optic nerve.

After chemotherapy containing vincristine, epirubicin, and cyclophosphamide and a subsequent therapy consisting of etoposide and ifosfamide, the patient underwent left skull base tumor resection and the tumor was completely resected. The postoperative pathology was consistent with ARMS (Figure 7D). Postoperative chemotherapy was continued with the regimen of etoposide and ifosfamide. After 2 weeks, CT and MRI imaging indicated recurrence (Figure 8). The patient received intensity-modulated radiotherapy (50 Gy 2 Gy/F/25F) and is still in follow-up now.

## Discussion

Due to the specificity of the growth site, nasal sinus ARMS often causes different clinical manifestations, such as headache, nasal congestion, and nasal bleeding. When the tumor invades the optic nerve or adjacent tissues, patients can present with corresponding symptoms such as diplopia, blindness, and difficulty in eye movement. Joo et al. (7) reported a case of ARMS that occurred in the right maxillary sinus with ipsilateral orbital invasion, causing proptosis and optic nerve involvement. Daniel et al. (8) reported a case of nasal sinus ARMS growing peripherally causing optic nerve invasion, resulting in the patient’s presentation of orbital apex syndrome with blindness as well as difficulty in eye movements. In the first case of this report, the patient had enlarged lymph nodes in the neck as the initial symptom, and the primary focus was found in the nasal sinuses after imaging inspections.

Based on histopathological features and molecular genetic characteristics, the World Health Organization (WHO) classified RMS into embryonal rhabdomyosarcoma (ERMS), ARMS, pleomorphic rhabdomyosarcoma, and sclerosing/spindle cell rhabdomyosarcoma in 2020 (9, 10). Approximately 70–90% of ARMS harbors specific genomic alterations such as *t(2;13) (q35;q14)* and *t(1;13) (q36;q14)* chromosomal translocations that produce *PAX3-FOX01* and *PAX7-FOX01* fusion genes, resulting in chimeric fusion protein alterations that drive transcription factor activation, thereby enhancing tumor cell proliferation and apoptosis (11). The diagnosis and differential diagnosis of ARMS depend on pathological features and immunohistochemical findings. Primitive small round tumor cells were arranged in the nest- and sheet-like structures, forming a characteristic alveoli-like structure, with fibrovascular intervals between the alveoli, partially showing skeletal muscle differentiation. According to the amount of fibrovascular interstitium, there are three tissue subtypes: classic, solid, and embryonic-alveolar mixed. The two cases we reported here were listed as classic ARMS, which showed distinct characteristic alveolar structures and no differentiation of rhabdomyoblasts. Myogenic markers such as MyoD1, desmin, and myogenin are usually positive in immunohistochemical detection (5).

Non-invasive imaging methods are important for the diagnosis of ARMS. CT can reveal the range of the disease and the invaded regions, such as the orbit, skull base, intracranial, infratemporal fossa, and other adjacent bones. Signs of calcification and hemorrhage are rare in head and neck RMS, while the bone destruction surrounding the tumor is relatively common (12). However, when the tumor and the inflammatory lesions in the sinus cavity similarly manifest soft tissue density, it is difficult to distinguish them. MRI can easily distinguish tumors and obstructive inflammation. The superior soft tissue resolution of MRI facilitates the assessment of detailed anatomical localization of soft tissue masses in the head, neck, and skull base and shows the involvement of adjacent areas (13, 14). The two cases we reported here showed a slightly low or low signal on T1WI, a high signal on T2WI, and a diffusion-limited high signal on DWI, which is correlated with the sparse cytoplasmic content of tumor cells and dense tumor cell growth. The edge of the lesion was clearer after contrast enhancement, and the lesions usually present with moderate or obvious enhancement, as well as circular inhomogeneous enhancement and progressive enhancement in the delayed phase (5, 15). ARMS on ^18^F-FDG PET/CT shows that the lesions with increased glucose metabolism and most malignant tumors occurring in the nasal sinuses also show increased uptake, the SUVmax of the two ARMS cases in our report ranged from 12.6 to 19.1. But in terms of differential diagnosis, previous literature reported that the SUVmax of primary sinonasal Non-Hodgkin’s lymphoma ranged from 2.0 to 36.4 (16), the SUVmax of sinonasal malignant melanoma ranged from 3.4 to 11.5 (17), and the SUVmax of inverted papilloma ranged from 2.0 to 36.4 (18). Based on the overlap of SUVmax ranges, SUV values have limitations in the differential diagnosis of malignant tumors occurring in the nasal cavity and sinuses. There is a need to provide more sample data in future studies to compare the differences in the performance of ^18^F-FDG PET/CT in different malignant tumors of the nasal sinuses. Even with some shortages, PET/CT can still comprehensively display the sites and extent of lesions and find the metastasis of lymph nodes and distant organs, especially in assessing lymph nodes and bone metastasis, which can effectively reduce missed diagnoses and provide evidence for clinical staging and treatment decisions. In this report, two patients were detected with more lesions by PET/CT. In addition, PET/CT can guide the selection of biopsy sites to improve the accuracy of biopsy based on the metabolic information (19–21). The combination of PET and MR (PET/MRI) has rapidly developed due to the superiority of PET in quantifying tracer metabolism and MRI in providing outstanding soft tissue contrast (22). There is still room for establishing the value of PET/MRI in clinical applications of ARMS, which will help to make more accurate diagnoses and improve the prognosis of patients.

The ARMS in the nasal sinuses needs to be differentiated from lymphoma, melanoma, and inverting papilloma. The nasal sinus lymphoma often arises in the anterior part of the nasal cavity or inferior turbinate, while the bone destruction is mild, permeable, and with bone contour, which typically showed “dashed” bone destruction. Lymphoma in MRI shows homogeneous isointensity or isosignal compared with brain parenchyma, DWI diffusion limitation, significantly reduced ADC value, and mild or moderate homogeneous enhancement after enhancement which is lower than that in turbinate mucosa (23). Melanoma of the nasal mucosa originates from melanocytes that migrate from the neural crest to the nasal cavity and nasal mucosa during the embryonic development period, mostly arising in the nasal cavity, with the middle and posterior segments of the nasal cavity frequently involved (24). Malignant melanomas containing more pigment appear as black or brown masses on endoscopic examination of the nose. Due to the melanin content, the characteristic performances on MRI include hyperintensity on T1WI, hypointensity on T2WI, mild to moderate heterogeneous enhancement, and adjacent bone destruction (25). Inverted papilloma mostly occurs in the lateral wall of the nasal cavity, with a predominantly expansive growth. The border is clear and the density is homogeneous on CT, and a sclerotic edge can be found at the base of the tumor. MRI demonstrates characteristic gyrus-like enhancement (26). Although imaging examination is of great value in localizing nasal sinus tumors, assessing the extent and involvement of adjacent structures, narrowing the differential diagnosis, and improving the accuracy of biopsy pathology and tumor staging, biopsy pathology is still the main basis for diagnosis, staging, and treatment planning.

Because of the aggressive behaviors and high degree of malignancy of ARMS in the nasal sinuses, it is usually advanced at diagnosis, and personalized treatment should be developed according to the patient’s specific situation. Currently, a multimodal treatment regimen combining surgery, chemotherapy, and radiation therapy is used to reduce the recurrence and metastasis rate (27–29). Chemotherapy includes preoperative and postoperative chemotherapy, and common drugs include vincristine, actinomycin D, ifosfamide, cyclophosphamide, and etoposide. Radiotherapy includes body surface irradiation and ^125^I-seeds implantation. Some literature reported that age, tumor size, aggressiveness, presence of distant metastases, regional lymph node involvement, and pathological response after chemotherapy were factors affecting the prognosis of ARMS (29, 30). Early diagnosis and standardized treatment are crucial for improving patient survival and prognosis. The overall survival of the first patient in this report was 9 months and the second patient is still being treated and followed up, and we cannot predict the final survival.

## Conclusion

In conclusion, the nasal sinus ARMS is highly malignant with a poor prognosis. Accurate diagnosis relies not only on histopathology and immunohistochemistry examination but also on genetic detection of characteristic chromosomal translocations and fusion genes. Imaging methods such as MRI and PET/CT can accurately assess the extent of the lesions and metastases, assist in the diagnosis of the disease and the selection of treatment regimens, provide precise localization for surgery, and help with treatment monitoring and follow-up.

## Data availability statement

The original contributions presented in the study are included in the article/supplementary material, further inquiries can be directed to the corresponding author.

## Ethics statement

The study was approved by the Institutional Review Board at the First Affiliated Hospital of Zhengzhou University and Peking University First Hospital. The participant gave written consent to participate in the study.

## Author contributions

WH: manuscript draft. YZ: acquisition and analysis of the study and imaging data collection and analysis. GG and LL: imaging data collection and analysis and resources. YQ and QY: formal analysis and resources. LK: supervision and writing—review and editing. All authors met the requirements for authorship for the submitted version and agreed to its submission.
